# Linking fathers and children in administrative data for public health research: A systematic scoping review.

**DOI:** 10.23889/ijpds.v7i3.2043

**Published:** 2022-08-25

**Authors:** Irina Lut, Katie Harron, Pia Hardelid, Margaret O'Brien, Jenny Woodman

**Affiliations:** 1 UCL ICH; 2 Thomas Coram Research Unit, Social Research Institute, UCL Institute of Education

## Objectives

The evidence-base for inter-related paternal and child health trajectories is not supported by linked, routinely collected administrative data in England. We conducted a scoping review to identify methods used globally for linking fathers and children in administrative data for public health research and map these methods onto dimensions of fatherhood.

## Approach

We searched PubMed, Scopus and Google/Google Scholar for articles published 2000-2020 from OECD countries that link fathers’ and children’s records in administrative data. Search terms included for example: “father” and “administrative data” or “record linkage”. The exposures of interest were any demographic, health, or other characteristics of fathers during their offspring's childhood or prior to birth captured within administrative data. We included studies where paternal information came from fathers' linked records and if outcomes were related to child health and development (e.g. educational attainment, behavioural outcomes, hospital admissions) measured before 18 years of age.

## Results

We identified 77 studies that quantified the association between paternal exposures and child health and development outcomes, using linked administrative data on fathers and their children. Four methods have been used globally to link fathers and children across vital statistics, health, social care, education, and criminal justice records. These methods are based on personal identity numbers (PINs), address or household identifiers, information from birth registrations, or health claims. We mapped what we can learn from these different linkages to dimensions of fatherhood identified through a conceptual framework. For example, for the linkage method based on address/household identifiers, co-residency may be used as a proxy for all dimensions of paternal involvement. However, substantial assumptions are needed when using linkage methods as proxies for paternal involvement.

## Conclusion

In many settings, changes in practice (such as recording fathers’ National Health Service numbers on hospital birth notifications in England) are required to facilitate linkage of father-child health data at the population level. This would help advance fatherhood research and enable targeted service offers to new fathers.

